# Activated Carbon in the Third Dimension—3D Printing of a Tuned Porous Carbon

**DOI:** 10.1002/advs.201901340

**Published:** 2019-08-09

**Authors:** Hendryk Steldinger, Alessandro Esposito, Kai Brunnengräber, Jan Gläsel, Bastian J. M. Etzold

**Affiliations:** ^1^ Ernst‐Berl‐Institut für Technische und Makromolekulare Chemie Technische Universität Darmstadt 64287 Darmstadt Germany

**Keywords:** 3D printing, activated carbon, CO_2_ activation, porogen templating, stereolithography

## Abstract

A method for obtaining hierarchically structured porous carbons, employing 3D printing to control the structure down to the lower µm scale, is presented. To successfully 3D print a polymer precursor and transfer it to a highly stable and structurally conformal carbon material, stereolithography 3D printing and photoinduced copolymerization of pentaerythritol tetraacrylate and divinylbenzene are employed. Mechanically stable structures result and a resolution of ≈15 µm is demonstrated. This approach can be combined with liquid porogen templating to control the amount and size (up to ≈100 nm) of transport pores in the final carbonaceous material. Additional CO_2_ activation enables high surface area materials (up to 2200 m^2^ g^‐1^) that show the 3D printing controlled µm structure and nm sized transport pores. This unique flexibility holds promise for the identification of optimal carbonaceous structures for energy application, catalysis, and adsorption.

## 1 Introduction

High surface area carbons are a key element in various applications, e.g., electrical^1^, ^2^ and solar energy conversion,^3^ energy storage,^4^, ^5^ gas separation^6^, ^7^ and storage,^8^, ^9^ waste water treatment,^10^, ^11^ and so on. Materials mainly used are activated carbons, carbon black, and carbon nanomaterial, which are obtained as powders. However, powders are disadvantageous in many applications as the low electrical conductivity induces ohmic losses in electrochemical applications and the low thermal conductivity can limit application, e.g., in adsorptive heat pumps. The high pressure drop of powder beds can also create additional energy consumption in continuous flow applications, such as redox flow batteries. These disadvantages can be overcome by employing structured materials, such as extruded monoliths, foams, or regular open cellular structures. While the advantage was proven, e.g., employing binders to obtain carbon/binder composite structures, monolithic polymers as precursor or monolithic hard templates, the ability to control the final shape is limited.^12^, ^13^, ^14^, ^15^

In contrast, extreme flexibility of structure could be obtained through 3D printing for many material classes like polymers,^16^, ^17^, ^18^ or metals^19^, ^20^ using various methods such as electron^21^ or laser melting,^19^, ^22^ fused filament extrusion,^18^ electrodeposition^20^ or stereolithography (SLA).^17^ For carbon, and especially porous carbon, this flexibility could only be leveraged to a limited extend as one of the major applied techniques—melt extrusion or beam melting—is not applicable to carbon directly. Also, the detour to first melt extrude polymers and subsequently pyrolyzing these to carbon is limited, as the polymers need to be thermoset in order to be structurally stable during pyrolysis, in contrast to thermoplastic polymers, which can be melt‐extruded for 3D printing. Due to this, 3D printing of carbon is a younger but recently very rapidly developing field. The major method applied up to now for obtaining 3D printed carbonaceous material is direct ink writing. By extruding an ink containing graphene oxide (GO), shaping, e.g., by ice templating and subsequent crosslinking of GO (e.g., through reduction) 3D printed carbons are accessible.^23^, ^24^, ^25^ In a similar approach, also a mixture of activated carbon and binder can be shaped by direct ink writing and pyrolyzed to obtain a carbon/activated carbon composite.^26^ The thixotropic rheology of some resorcinol/formaldehyde inks was employed to direct ink write resorcinol/formaldehyde structures, which resulted after polymerization and pyrolysis in carbonaceous material.^27^ These achievements, on the one hand, showed the great possibilities that get accessible through controlling the macroscopic structure of carbonaceous material through 3D printed, e.g., for catalysis,^28^ CO_2_ capture,^26^ microbial fuel cells,^29^ or energy storage.^24^, ^27^ On the other hand, the materials obtained up to now have limitations in the control of porosity below 1 µm, especially micro‐ and mesoporosity and show mainly low specific surface area. This porosity is very often generated in an uncontrolled manner, e.g., during the pyrolysis of the binder. Thus, it remains a challenge to 3D print truly tailored hierarchically structured materials. Furthermore, as for direct ink writing, the key to successfully obtaining decent structures is the identification of a proper ink and suitable nozzle, the flexibility in different scales of resolution as well as in materials is limited.

As an alternative, SLA allows 3D printed, cross‐linked and thermosetting polymers, through layer‐by‐layer photopolymerization of a monomer resin with a higher resolution than extrusion based techniques.^30^ Very recently, two reports demonstrated that SLA printing of polymers (also GO dispersion) and subsequent pyrolysis is a suitable alternative to obtain 3D printed carbons.^29^, ^31^ The studies show that also with this approach no proper control of the porosity below 1 µm and especially of micro‐ and mesoporosity is accessible.

In this work, we propose that the reason for the lack of porosity control is the instability of commercial SLA photoresins, which are acrylate based. These tend to depolymerize at high temperatures, which can lead to uncontrolled micro‐ and mesoporosity within the final carbon.^32^

**Figure 1:**
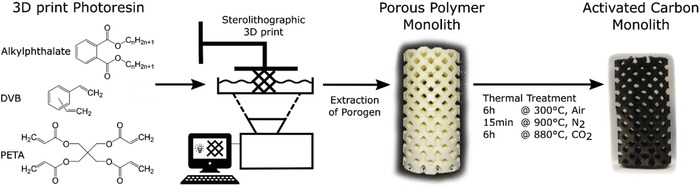
Schematic overview of the 3D printing process, starting from the liquid photoresin, then producing a porous polymer open cell structure (tetragonal unit cell) by stereolithographic 3D print and subsequent extraction of the porogen phase, finally yielding an activated carbon open cell structure upon a thermal treatment consisting of stabilization in air, pyrolysis in nitrogen and activation in CO_2_.

In this work, the limitation is overcome through photoinduced copolymerization of the monomers pentaerythritol tetraacrylate (PETA) and divinylbenzene (DVB) during SLA printing. Tuning the porosity of hierarchically structured materials on several scales is achieved through a multiple‐step synthesis (**Figure** **1**) where during the first step of SLA, the macroscopic structure is controlled on the mm and µm scale. Liquid porogen templating during SLA printing is used to add meso‐ and macropores. In porogen templating, an inert porogen is dissolved in the monomer starting mixture and during polymerization, the porogen solubility decreases, resulting in phase separation.^33^, ^34^ This porogen phase acts as a template, which is removed in the second step, extraction. In order to maintain the macro‐ and microstructure during pyrolysis, the polymer is stabilized at the third step by oxygen curing and then pyrolyzed at the fourth step. In the final CO_2_ activation, the microporosity of the resulting carbon structure can be increased.

## 2 Results and Discussion

### 2.1 Identifying a Suitable Photoresin Mixture

A photoresin mixture employed for stereolithography contains monomer as the major component, as well as an initiator to start polymerization at illuminated spots, and a stabilizer to prevent polymerization at nonilluminated spots. A dye can also be part of the mixture to control light penetration depth. While several commercially available and research based mixtures can be used to obtain SLA printed polymers, in this work the resulting polymer structure needed to be converted to a carbon structure by pyrolysis. During conversion, a sufficient carbon yield should result and the original structure, besides conformal shrinkage, should be retained. Another major component of the photoresin mixture used here is the porogen, which should not participate in photopolymerization, but result in phase separation and pore template formation.

As it is a major component, a suitable monomer needs to be identified first. For two acrylic‐based monomers (pentaerythritol tetraacrylate—PETA; poly(ethylene glycol)diacrylate—PGD) and one aromatic monomer (divinylbenzene—DVB), the two key indicators of i) solidification time during photopolymerization and ii) carbon yield after stabilization and pyrolysis were assessed. Besides the varied monomer, 50 vol% of porogen was added, as it could influence the solidification time and carbon yield due to being the second major component.

**Figure 2:**
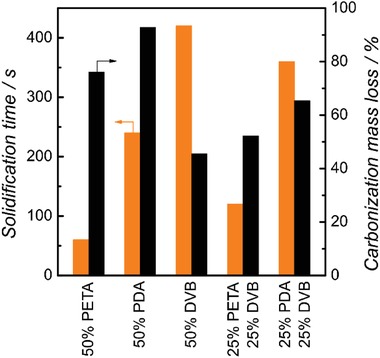
Comparison of solidification times of photoresins, consisting of 50% DEP, 50% monomer and 10 mg mL^‐1^ BAPO as initiator, under an HPHG lamp and mass loss during oxidation and carbonization of the corresponding porous polymers.

**Figure** **2** shows the resulting solidification time and carbon yield obtained for the three monomers. The aromatic DVB took around 420 s to harden, the acrylic PDA 240 s and PETA 60 s. Radical stabilization in the sp²‐hybridized system of the DVB molecule is responsible for the low reaction speed of aromatic monomers, which would result in impracticable 3D printing times.^35^ The acrylates reacted much faster; among them, PETA had the fastest solidification time as the tetrafunctional PETA needs a lower conversion of the vinyl groups to obtain stable structures^36^ compared to the difunctional PDA monomer. Nevertheless, both acrylic homopolymers show a very low carbon yield of 7% and 22% after pyrolysis. At these high mass losses, the polymer precursor is not used efficiently, which results in very fragile carbon structures and high shrinkage.

In contrast, DVB‐based structures show a carbon yield of 54%. This can be attributed to the thermal instability of the acrylate group and the possibility of stabilization of the aromatic structure by oxygen crosslinks, as shown in the literature.^37^ Based on this, none of the three monomers suits the envisaged synthesis strategy. A copolymer, consisting of an acrylate‐based monomer and an aromatic monomer, could combine the advantages of both monomer classes. The solidification time and carbon yield were studied for volumetric 1:1 mixtures of PETA:DVB and PDA:DVB (plus the 50 vol% of the porogen). As shown in Figure 2, combining both monomers clearly yields synergistic effects. The DVB:PETA mixture had a solidification time of 120 s combined with a high carbon yield of 48%.

### 2.2 Copolymerization of PETA/DVB

While the above shows that a mixture of PETA and DVB appears capable of yielding carbons in suitable photopolymerization time, it must be ensured that true copolymerization of both monomers takes place during photopolymerization within the stereolithography 3D printer. Only in true copolymerization is the slowly reacting DVB inserted homogeneously into the copolymer chain. Parallel homopolymerization of both monomers is not desired as it would result in separate polymer phases with different decomposition behavior upon pyrolysis.

**Figure 3:**
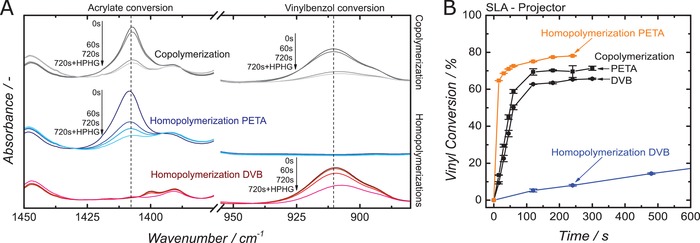
A) IR Spectra of photoresins containing 50% DEP as porogen and either 50% DVB, 50% PETA, or 25% DVB plus 25% PETA, after different illumination times in the 3D printer; B) Conversion of aromatic and acrylic vinyl groups as a function of illumination time in the 3D printer for the homopolymerizations of DVB or PETA and the copolymerization of DVB and PETA together with 50 vol% DEP as porogen.

To obtain insights into whether sufficient copolymerization takes place, the vinyl group conversion was detected via IR spectroscopy for different illumination times within the SLA 3D printer (**Figure** **3**A). The strong CH_2_ scissoring band at 1408 cm^−1^, belonging to PETA, can be clearly separated from the weak DVB adsorption peak at 1400 cm^−1^. CH_2_ wagging at 907 cm^–1^ can be clearly attributed to DVB while the porogen is not contributing at these wavelengths.^38^ The reaction progress of the PETA and DVB monomer was deduced from these bands. Figure 3B displays the reaction progress for the individual monomers and the monomer mixture, which always contains DEP as porogen (see Figure S1, Supporting Information for the full polymerization time and kinetics using DIP as porogen). Similar to the solidification time experiments, the IR study of individual monomer polymerization reveals an orders of magnitude higher consumption rate for PETA, compared to DVB. Interestingly, employing the mixture of PETA and DVB results in the same consumption rate for both monomers. This is a strong indication that a true copolymerization takes place and the unwanted parallel homopolymerization of the monomers is suppressed. Copolymerization needs 120 s for ≈65% degree of conversion of vinyl groups for both monomers to be achieved; this is a reasonable amount of time for SLA 3D printing.

### 2.3 Vertical Resolution and Printing Speed

**Figure 4:**
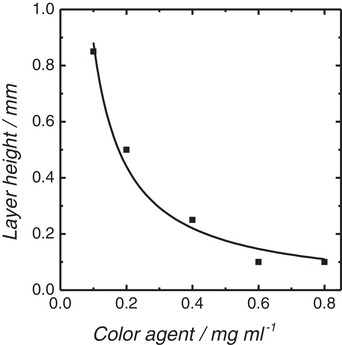
Single 3D printed layer thickness produced from resin containing 35% DVB, 35% PETA, 30% DIP as a function of the dye concentration.

**Figure 5:**
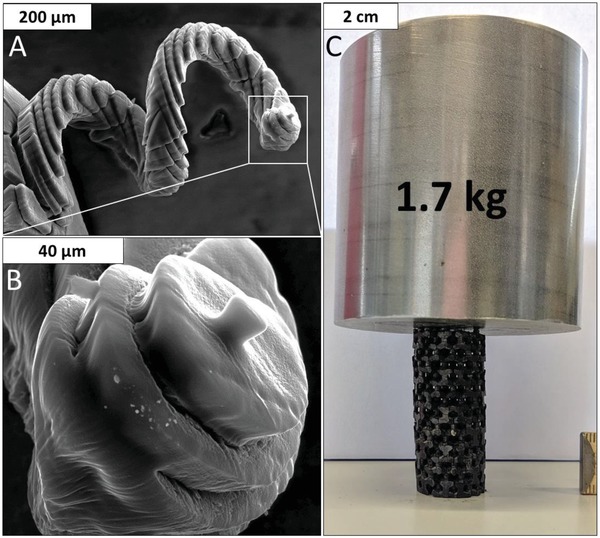
A,B) SEM images of a carbon spiral that was 3D printed with a dye concentration of 0.8 mg mL^−1^ and a layer thickness of 20 µm; C) Carbon open‐cell structure based on a tetrahedral unit cell and 3D printed with a dye concentration of 0.4 mg mL^−1^ and a layer height of 100 µm supporting the weight of a 1.7 kg steel cylinder.

Vertical resolution during stereolithography can be adjusted through addition of a dye, as high absorption of the illuminating light leads to very thin layers. Thin layers or very high vertical resolution also results in very slow printing. Depending on the structure to be printed, the optimal vertical resolution and dye concentration needs to be known. Sudan1 was chosen as dye as it adsorbs in the emission spectrum of the adsorption spectrum of the photoinitiator (see Figure S2 in the Supporting Information). To study the influence of the dye concentration, the resulting layer thickness after 120 s of illumination was determined for dye concentrations varying from 0.1 to 0.8 mg mL^−1^. As shown in **Figure** **4**, the layer thickness decreases from 0.85 to 0.1 mm with increasing dye concentration. This trend can be described well with the Beer‐Lambert Law when accounting for the mixed absorption of the dye and photoinitiator. These insights were employed to study the maximum achievable vertical resolution and printing speed. The commercial printer has a minimum step width of 5 µm and in‐plane pixel resolution of 25 µm. Based on this, the minimum layer height 20 µm was chosen to be printed with a dye concentration of 0.8 mg mL^−1^, and the maximum layer height 400 µm at a dye concentration of 0.1 mg mL^−1^. The test structures printed were spirals of different sizes and an open cell structure based on a tetrahedral unit cell. **Figure** **5** comprises photographs and SEM images of the stabilized and pyrolyzed structures. The smallest spiral printed, using the high dye concentration of 0.8 mg mL^−1^, featured a thread diameter of 135 µm for the polymer and 85 µm after stabilization and pyrolysis. The smallest detail of this carbon spiral was a single dot at the top of the structure with a size of 15 µm. This is similar compared to other stereolithography methods^31^ and shows the advantage compared to extrusion based 3D printing processes. However, employing this high vertical resolution has the disadvantage of slow printing speed, which is 6 µm min^−1^, as the processing time per layer is constant regardless of its thickness. A much higher printing speed of 118 µm min^−1^ resulted for the 400 µm step height and dye concentration to 0.1 mg mL^−1^ leading to a coarser resolution. For the printing of larger structures, such as the open cell structure shown in Figure 5C, an intermediate dye concentration of 0.4 mg mL^−1^ was found to be suitable, as it combines lower printing times with acceptable accuracy. Advantageous is the broad range of resolution easily accessible through the SLA approach, which is limited for direct ink writing to the nozzle employed.

While mechanical stability is not within the focus of this study, it can be stated that all structures produced could be handled without special care. As an initial quick test for mechanical stability a weight of 1.7 kg was placed on a carbon open cellular foam printed (see Figure 5C), which did not result in any structural damage. Based on the real front end surface area a minimal crushing strength of 100 kPa can be stated. Nevertheless, as the structure was not stressed until crushing the real crushing strength may be pronounced higher. Regarding the sizes of the carbonized structures, shrinkage throughout the pyrolysis process has to be taken into account. For the resin mixture employed, a reduction of 35% in each dimension was observed, which is significantly lower than values of 62%^29^ and 66%^39^ reported in the literature, which shows that the polymer and polymer–graphene composite do not undergo stabilization prior to pyrolysis, which resulted in distortion of the printed specimen. In contrast, the method presented here allows for conservation of small details in printed parts. Some surface cracks were observed though they did not lead to mechanical instability of the structures.

### 2.4 Porosity Control Through Porogen Templating

The structures discussed previously were printed with a resin mixture containing porogen. This section examines extraction of the porogen and how porogen amount and type (different alky chain lengths) influences the final pore structure.

Studying the mass loss after extraction (see Figure S3 in the Supporting Information) demonstrates that full extraction of the porogen is possible for samples containing 20 vol% or more of porogen in the green body.

**Figure 6:**
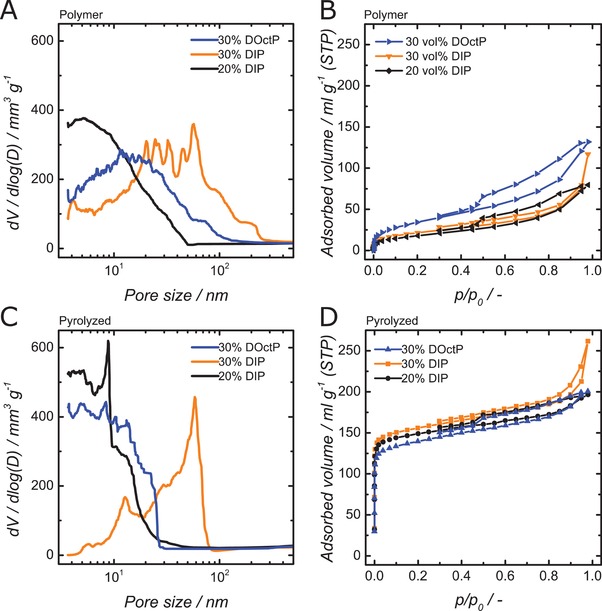
A) Pore size distributions from HG porosimetry of extracted polymers; B) N_2_ sorption isotherms of extracted polymers; C) Pore size distributions from HG porosimetry of pyrolyzed carbons; D) N_2_ sorption isotherms of pyrolyzed carbons.

To examine the influence of porogen type and content on the pore structure after porogen extraction, stabilization and pyrolysis, N_2_ sorption and mercury intrusion porosimetry were conducted. The pore structure of the polymers after porogen extraction is discussed. Hg porosimetry delivers the pore size distribution of the meso and macropores (see **Figure** **6**A and **Table** **1**). Employing 30 vol% DIP as porogen results in the largest pores and a maximum pore diameter (expressed by *d*_90%_) of 155 nm. A reduction in the porogen volume fraction to 20% reduces the pore sizes significantly, yielding a maximum pore size of 30 nm. Pore size reduction can also be achieved at the higher porogen volume of 30% when reducing the alkyl chain length of the porogen from C_10_ (DIP) to C_8_ (DOctP): a maximum pore diameter of 80 nm results. The TEM images given in Figure S4 (Supporting Information) corroborate these findings and show within unstructured carbon irregular voids within these size ranges. These observations of the influence of porogen amount and type agree with results from suspension polymerization in the literature.^40^ N_2_‐physisorptions of the porous polymers show only a minor number of micropores (see Figure 6B and Table 1). For mesoporosity, the sorption analysis agrees with the findings of the Hg porosimetry and the general trends in porogen type and amount. The specific surface areas (BET) of the resulting porous polymers range from 64 to 125 m^2^ g^−1^.

**Table 1:** Burn‐off during pyrolysis, BET analysis results for the polymers, and QSDFT analysis results for the pyrolyzed carbons from N_2_ sorption analysis, and the maximum pore diameter derived from the Hg porosimetry for the polymers and pyrolyzed carbons

Porogen	Burn off	Nitrogen sorption				Hg porosimetry	
	Pyrolysis	Polymer	Pyrolyzed carbon			Polymer	Pyrolyzed carbon
	∆*m* [%]	SSA (BET) [m^2^ g^−1^]	SSA (QSDFT) [m^2^ g^−1^]	*V*_Pore_ [cm^3^ g^−1^]	*d*_Pore_ [nm]	*d*_90%_ [nm]	*d*_90%_ [nm]
20 vol% DIP	66.6	64	601	0.28	0.75	30	28
30 vol% DIP	69.8	77	655	0.35	0.72	156	69
30 vol% DOctP	68.4	125	536	0.29	0.81	81	26

Hg‐porosimetry‐based pore size distributions and N_2_‐physisorption isotherms resulting from stabilization and pyrolysis are given in C and D, respectively. The results are summarized in Table 1. Compared to the porous polymer starting material, the pore sizes decrease, which most likely results from the material shrinking. The general trend of the porogen type and content influencing pore sizes also applies to the carbon structure. The N_2_‐physisorption results show that during pyrolysis, additional microporosity is introduced while meso‐ and macroporosities were retained. The resulting micropore structure should be mainly influenced by the copolymer and pyrolysis conditions, not by the extracted porogen. This is confirmed by the similarity of the sorption isotherms for different porogens and surface areas ranging from 536 to 655 m^2^ g^−1^.

### 2.5 Increasing Microporosity Through CO_2_ Activation

**Figure 7:**
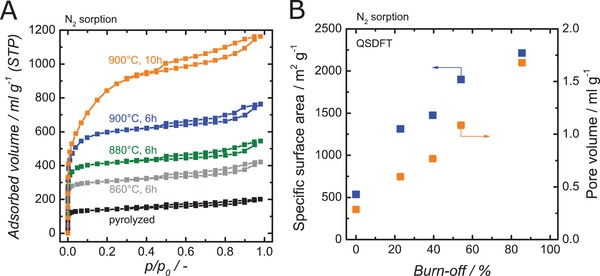
A) N_2_ sorption isotherms of 3D printed carbon templated with 30 vol% DOctP after pyrolysis and CO_2_ activated at temperatures ranging from 860 to 900 °C; B) SSA and total pore volume derived with QSDFT as a function of burn off through CO_2_ activation of 3D printed and pyrolyzed carbon (30 vol% DOctP).

While the pyrolyzed structures show some microporosity, the specific surface areas might be too low for applications like adsorption or catalysis. Therefore, the effect of increasing the specific surface area with an additional CO_2_ activation step was studied. Varying the activation temperature from 860 °C to 900 °C and time from 6 to 10 h enabled the burn off to be adjusted from 23% to 85%. All activated structures were stable and could be handled normally after CO_2_ activation. **Figure** **7** and **Table** **2** summarize the results of the sorption characterization. The nonactivated carbon has a specific surface area of 536 m^2^ g^−1^ and a pore volume of 0.29 mL g^−1^; at the highest burn off (85%), a pore volume of 1.68 mL g^−1^ and QSDFT‐based specific surface area up to 2200 m^2^ g^−1^ results, which is much higher than generally reported for 3D printed carbon.^27^ Both parameters depend linearly on burn off (Figure 7B) and can thus be adjusted well. At the smallest degree of activation, the average pore size decreases from 0.81 to 0.75 nm, which stems from the opening of clogged small micropores. At higher degrees of activation, the average pore size increases up to 1.3 nm for the maximum burn‐off due to widening of the micropores.

**Table 2:** Mass loss and calculated data (BET and QSDFT) from nitrogen sorption measurements of CO_2_ activated carbon (30 vol% DOctP)

Activation method	Burn off	Nitrogen sorption			
	∆*m* [%]	SSA (BET) [m^2^ g^−1^]	SSA (QSDFT) [m^2^ g^−1^]	*V*_Pore_ [cm^3^ g^−1^]	*d*_Pore_ [nm]
Nonactivated	–	538	536	0.29	0.81
860 °C, 6 h	22.9	1200	1312	0.60	0.75
880 °C, 6 h	39.5	1609	1474	0.77	0.83
900 °C, 6 h	54.1	2247	1898	1.08	0.96
900 °C, 10 h	85.5	3019	2213	1.68	1.27

## 3 Conclusions

This work demonstrates a novel method of obtaining tunable porous carbon structures. Photoinduced copolymerization of PETA and DVB was used for SLA 3D printing, which showed that it is possible to combine fast printing with high final carbon yield, properties not achievable when employing monomers despite copolymerization. The full flexibility of 3D printing of computer designed structures can be leveraged with this approach, enabling unprecedented resolution of the final carbon structure down to ≈15 µm. The advantages of SLA 3D printing can be combined with porogen templating without comprising the printing resolution. Porogen templating allows addition of transport pores of varying size (up to ≈100 nm) and amount to the final carbon structure. Additional CO_2_ activation enables tuning of the micropore content, resulting in a specific surface area of up to 2200 m^2^ g^–1^. Porous properties and versatility of conventional activated carbons were combined with the ability to freely design high‐resolution 3D printing with controlled channels in the µm regime. This new and flexible approach to hierarchically structured carbon materials paves the way to identifying and applying optimized carbon materials in various applications. Applications in which the connected structure can be leveraged, e.g., due to higher electrical or thermal conductivity, stand to benefit: computer optimized electrodes in electrochemical applications are a very promising field.

## 4 Experimental Section

*Resin Preparation*: Three different monomers were used for the initial evaluation of solidification time. Poly(ethylene glycol)diacrylate (PDA, *M*_n_ = 575 g mol^−1^, 400 to 600 ppm 4‐methoxyphenol), pentaerythritol tetraacrylate (PETA, containing 10–40% triacrylate, 350 ppm hydroquinone) and divinylbenzene (DVB, containing 20% ethylstyrene, 1000 ppm p‐*tert*‐butylcatechol) were purchased from Sigma Aldrich and used without extracting the stabilizer. The initiator phenylbis(2,4,6‐trimethylbenzoyl) phosphine oxide (BAPO, 97%), the color agent sudan1 (≥95%), and dibutyl phthalate acting as porogen (DButP, 99%) were also bought from Sigma–Aldrich. The porogen bis(2‐ethylhexyl) phthalate (DOctP, ≥98%) was purchased from Alfa Aesar and the porogen diisodecyl phthalate (DIP, ≥99%) from Merck. To prepare the photoresin, the monomers and porogen were mixed by shaking until homogeneous. The photoinitiator (and, in the case of 3D printing, experiments the dye) was added and shook until a homogeneous phase resulted.

*Photopolymerization*: To estimate the solidification time of different photoresin mixtures, in a preliminary study a high pressure mercury lamp (ULTRA‐VITALUX from Osram, 300 W) at a distance of 20 cm was employed for illumination.

To determine the single layer height, the photoresin (35% PETA, 35% DVB and 30% DOctP) was illuminated in the 3D SLA printer with a 30 mm × 0.5 mm rectangle image for 120 s.

For the 3D SLA printing, a Titan 2 HR printer from Kudo 3D was employed. To modify the 3D printing setup, a blue light filter (Schott BG‐3) was introduced between the DLP projector and the resin vat. An illumination time of 120 s, lifting speed of 4 mm min^−1^ lifting height of 4 mm, and a lowering speed of 10 mm s^−1^ were employed for the printing of the spirals and small open cell structures composed of 8 tetragonal cubic centered unit cells with a unit cell diameter of 5.7 mm and a thread diameter of 2 mm. Larger structures were printed using a reduced illumination time of 75 s.

From the Beer‐Lambert Law, the layer thickness as a function of the color concentration can be calculated (see Supporting Information for derivation of the formula):

(1) $$d_{\text{Layer}}=\frac{\frac{E_{\text{Poly}}}{\varepsilon_{\text{Sudan}1}}}{c_{\text{Sudan}1}+\frac{\varepsilon_{\text{Sudan}1}}{\varepsilon_{\text{BAPO}}}\cdot c_{\text{BAPO}}}$$

*Porogen Extraction*: After 3D printing, the porogen and color agent were extracted using soxhlet extraction with acetone (>20 mL g_Polymer_^−1^) for 24 h then dried at 60 °C over night.

*Oxygen Stabilization, Pyrolysis and CO_2_‐Activation*: The extracted 3D printed polymer was first oxidized in air for 6 h at 300 °C with a heating ramp of 10 K min^−1^. Pyrolysis of the oxidized sample was then conducted under nitrogen at a temperature of 900 °C for 15 min after heating at a rate of 3.3 K min^−1^. The carbon yield was calculated as the fraction of mass after oxidation and pyrolysis, divided by the mass of the extracted polymer. The samples were activated under CO_2_ at a temperature of 860 to 900 °C for 6 to 10 h after heating at a rate of 10 K min^−1^.

*IR Spectroscopy*: The degree of conversion of vinyl groups was tested by Fourier‐transform infrared spectroscopy. A 10 µm thick film was enclosed between two NaCl round crystal windows. For each data point, four measurements were made, with a 90° rotation to correct for thickness variation. As an internal standard, the alkyl chain adsorption represented by the adsorption maximum between 730 and 770 cm^−1^ was used. The maximum between 900 and 920 cm^−1^ accounted for the aromatic vinyl groups. In the range of acrylic vinyl groups, peak overlay occurred, so their concentration was determined from the difference between the minimum in a range of 1430 and 1440 cm^−1^ and the maximum between 1403 and 1420 cm^−1^. In these measurements, the 3D printer was used for illumination.

*Porosity Characterization*: Nitrogen sorption at a temperature of 77 K was conducted using a Quantachrome Quadrasorp apparatus. The samples were outgassed at 350 °C (carbons) or 150 °C (polymers) for 20 h until a minimum pressure of 14 mTorr was reached. The average micropore diameter was calculated using the formula for slit pores and QSDFT evaluation for the microporous pressure range 0–0.4 only. BET analysis was conducted for polymers. For N_2_ sorption, despite optimizing analysis parameters like equilibrium time and tolerance, the hysteresis loop could not be fully closed for all samples. This effect was often seen in specimens with intense diffusion limitation and could be attributed to micropores only accessible at higher pressures and pore filling.^41^ This effect was prominent among the pyrolyzed samples (not activated) that underwent a shrinking of 30% during the carbonization and might contain closed‐up micropores.

Mercury porosimetry was conducted using a combined Pascal 140 and Pascal 440 from Thermo Scientific in a range of 0.0125 to 400 MPa. Pore sizes were calculated using a cylindrical and plate pore model. In order to quantify the maximum pore size, the *d*_95_ value was used. At this value, smaller pores represent 95% of the pore volume.

*TEM Imaging*: Samples were prepared by dispersing a small amount of the sample material in 1.5 mL of ethanol utilizing an ultrasonic vial tweeter. The resulting black dispersion was diluted until it was slightly transparent and dispersed a second time in the vial tweeter. The dispersion was then allowed to settle for a few minutes. Afterwards a droplet of the dispersion was applied to a holey carbon grid and allowed to dry. High‐resolution TEM images were recorded using a JEOL JEM2100F (JEOL) with a field emission gun operating at a nominal acceleration voltage of 200 keV.

*SEM Imaging*: Field emission scanning electron microscopy images were taken using a Philips XL30 FEG. Polymer samples were sputtered with gold for 300 s at a potential of 30 mV. Pictures of polymers and carbon were taken at an acceleration voltage of 10 and 30 kV, respectively.

## Conflict of Interest

The authors declare no conflict of interest.

## Supporting information

Supplementary
