# Supplementary material for: Activated Carbon in the Third Dimension—3D Printing of a Tuned Porous Carbon
Source: Adv Sci (Weinh). 2019 Aug 9;6(19):1901340. doi: 10.1002/advs.201901340 (PMC6774063; doi:10.1002/advs.201901340)
Supplement: Supplementary file 1 — Supplementary [file ADVS-6-1901340-s001.pdf]

Copyright WILEY-VCH Verlag GmbH & Co. KGaA, 69469 Weinheim, Germany, 2019.

ADVANCED SCIENCE
Open Access

Supporting Information

for *Adv. Sci.,* DOI: 10.1002/advs.201901340

Activated Carbon in the Third Dimension—3D Printing of a Tuned Porous Carbon

*Hendryk Steldinger, Alessandro Esposito, Kai Brunnengräber, Jan Gläsel, and Bastian J. M. Etzold**

Copyright WILEY-VCH Verlag GmbH & Co. KGaA, 69469 Weinheim, Germany, 2019.

Supporting Information

Activated carbon in the 3rd dimension - 3D printing of a tuned porous carbon

*Hendryk Steldinger, Alessandro Esposito, Kai Brunnengräber, Jan Gläsel and Bastian J.M. Etzold**

1. Monomer conversion

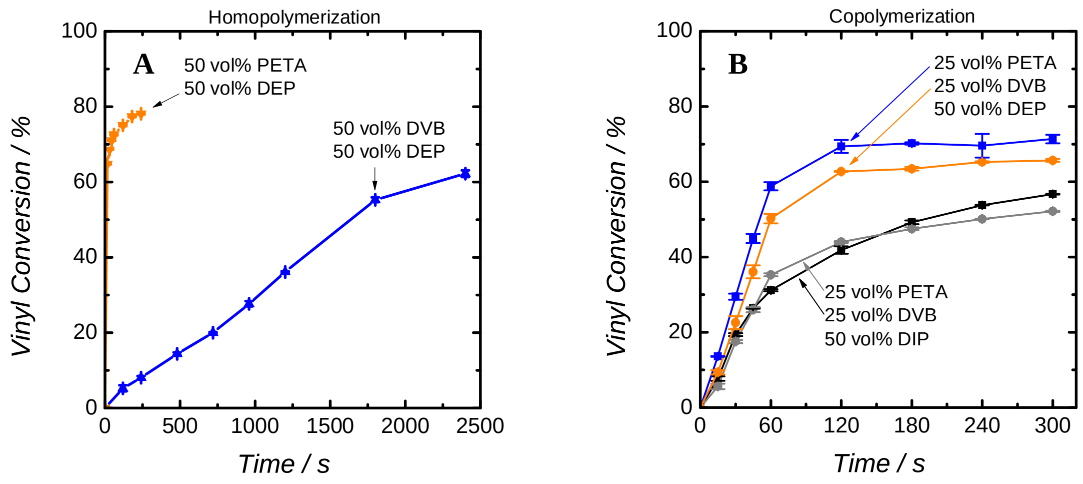

Figure **S1** A and B show additional information concerning the polymerization kinetics of the homopolymerizations and copolymerizations respectively. It illustrates that, despite similar reaction rates at the beginning of the reaction (< 30 s), in case of DIP as porogen a drop in reaction rate compared to the resin containing DEP becomes apparent. However, after 120 s of illumination with the 3D printer or the mercury lamp both resins solidify.

2. Layer height

In order to examine the relationship of layer height and color concentration, a regression analysis employing the Beer-Lambert Law was conducted. Here, the derivation of this equation for the calculation of the layer height d_{Layer} is shown. The Beer-Lambert Law shown in eq. (S1) describes the energy dose of light utilized for the polymerization E_{Poly} as a function of the layer height and

concentrations c together with the extinction coefficients ϵ of the dye Sudan 1 and the photoinitiator BAPO.

$$E_{Poly} = d_{Layer} \cdot (\epsilon_{Sudan1} \cdot c_{Sudan1} + \epsilon_{BAPO} \cdot c_{BAPO}) \qquad \text{(S1)}$$

Knowing that a constant energy dose is required for the polymerization, the layer height can be expressed as a function of the dye concentration as shown in eq. (S2). Of course, when printing more complex structures the energy dose may change due to temperature change, build up of oligomers in the resin or consumption of photoinhibitor.

$$d_{Layer} = \frac{\frac{E_{Poly}}{\epsilon_{Sudan1}}}{c_{Sudan1} + \frac{\epsilon_{Sudan1}}{\epsilon_{BAPO}} \cdot c_{BAPO}} \qquad \text{(S2)}$$

3. UV-VIs spectra of light source and dye

Figure S2 displays the emission spectra of the projector with and without a blue light filter (Schott BG-3). The filter blocks out a wavelengths above 450 nm. The photoinitiator BAPO absorbs wavelengths up to 420 nm. Sudan1 as the dye is able to filter out the whole emission spectrum utilized for the polymerization.

4. Extraction of the porogen

In Figure S3, the reduction of the specimen mass is shown as calculated from the porogen mass and as measured reduction of mass. For the interpretation, it has to be taken into account that after the 3D print the specimen were directly extracted without drying. Therefore, residual resin on the surface and in the voids of the macrostructure contributes to the mass reduction as well resulting in higher mass losses measured.

5. TEM images of resulting carbon structure

In Figure S4 TEM images of a resulting carbon (using 30 vol % of DOctP as porogen and applying 10 h of CO_2 activation at 900 °C) are given. A void approx. 6.5 x 13 nm is seen, which gives qualitative insights into the irregular mesoporosity

6. Scanning electron micrographs of porogen templated carbon surface

In Figure S55, scanning electron micrographs of the surface of the 3D printed carbon templated with different porogens are displayed.

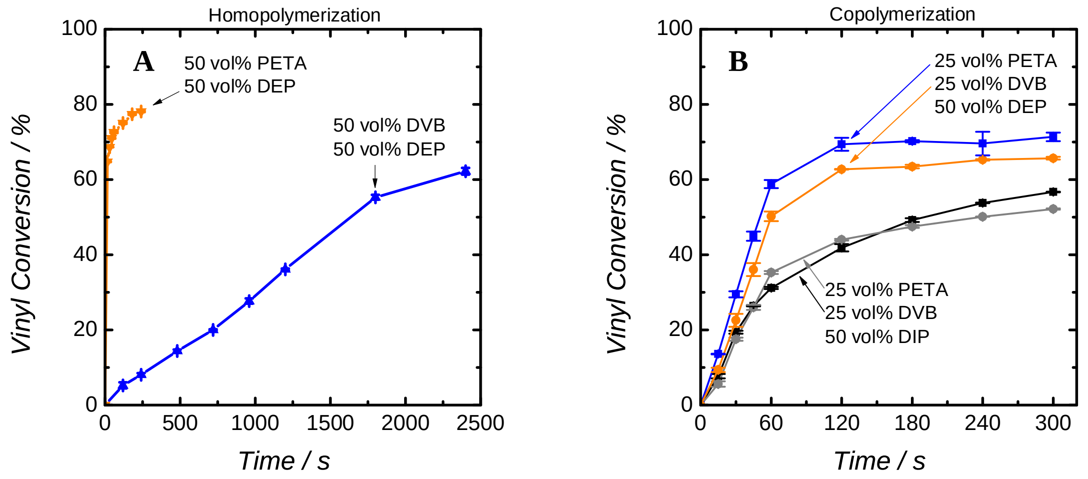

Figure S1: Conversion of aromatic or acrylic vinyl groups as a function of illumination time in the 3D printer in case of a homopolymerizations of DVB or PETA (A) or a copolymerization of DVB and PETA together with 50 vol% DEP or DIP as porogen (B).

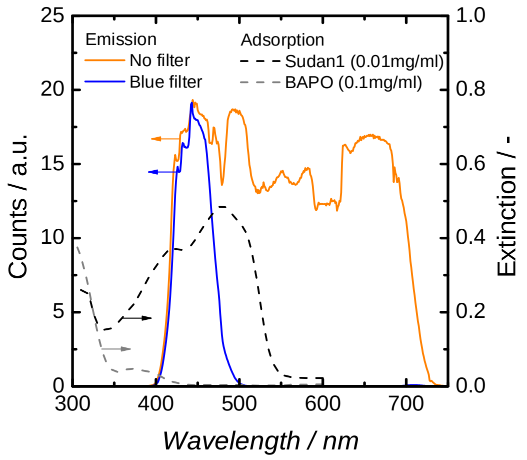

Figure S2: Emission spectrum of the 3D printer's projector (Optoma HD142X) in pristine condition and with blue glas filter and extinction of the photoinitiator BAPO and color agent Sudan1.

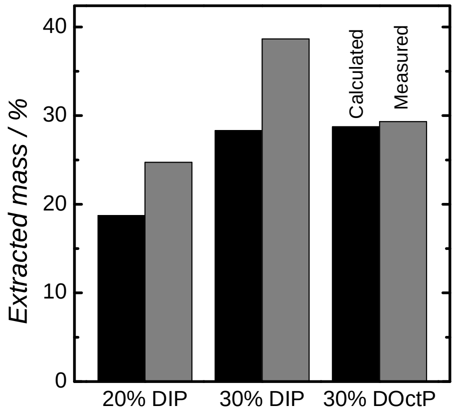

Figure S3: Calculated and measured extracted porogen mass upon soxhlet extraction with acetone.

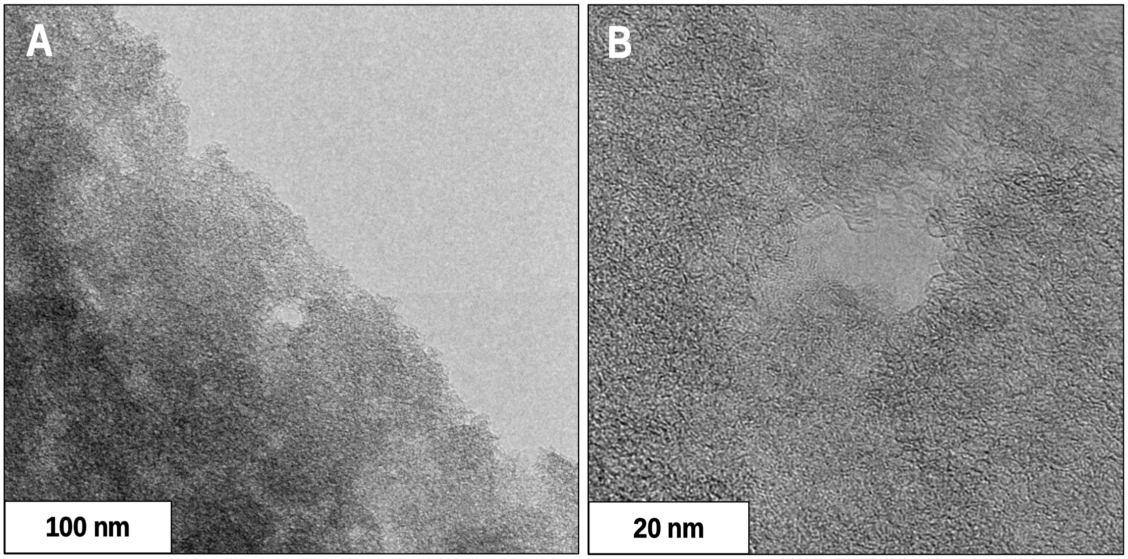

Figure S4: TEM image of 3D printed carbon (30 vol % DOctP; 10 h CO_2 @ 900 °C).

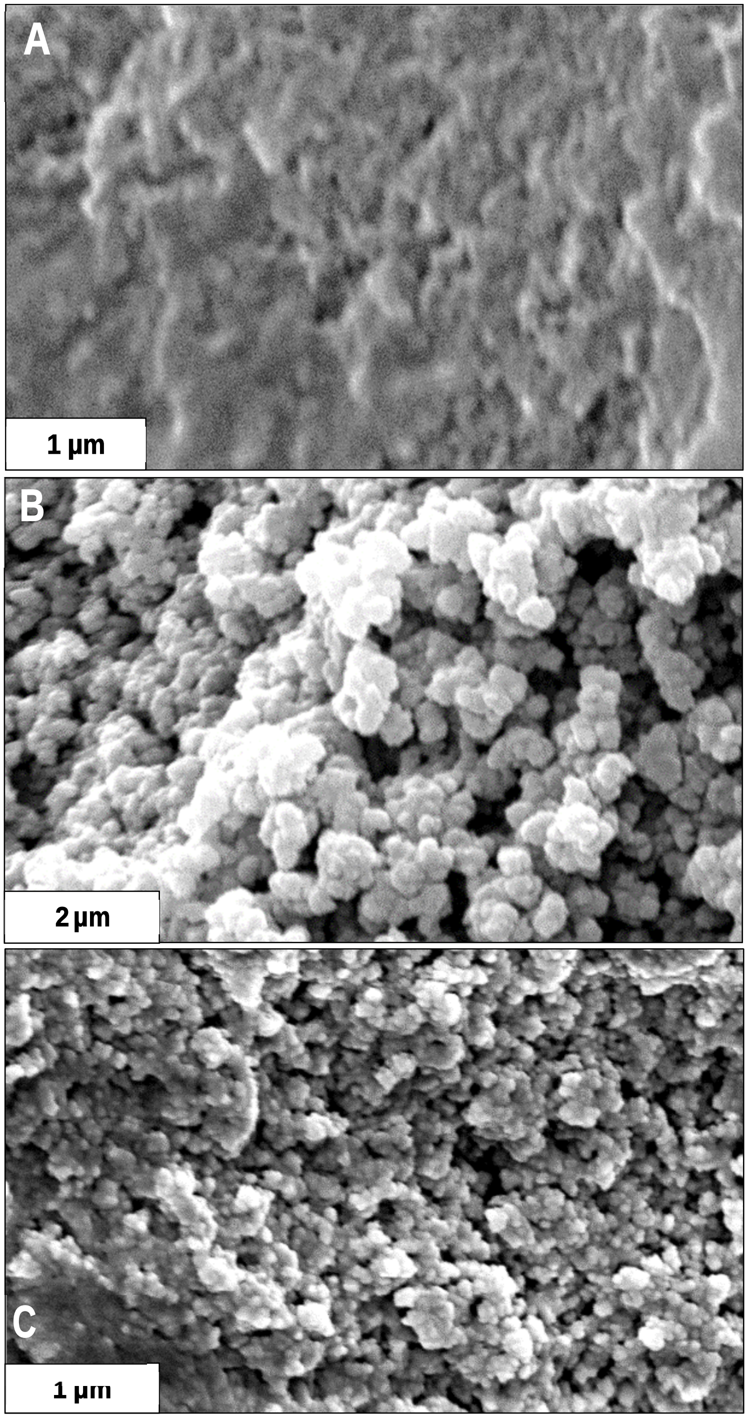

Figure S5: Surface micrographs of 3D printed carbon templated with 30 vol% DOctP (A), 30 vol% DIP (B) and 20 vol% DIP.
